# Endurance exercise training restores diabetes-induced alteration in circulating Glycosylphosphatidylinositol-specific phospholipase D levels in rats

**DOI:** 10.1186/s13098-020-00553-z

**Published:** 2020-05-19

**Authors:** Farzad Abdolmaleki, Ali Heidarianpour

**Affiliations:** Department of Exercise Physiology, Faculty of Sport Science, Bu-Ali Sina University, Hamedan, Iran

**Keywords:** Exercise training, Glycosylphosphatidylinositol-specific phospholipase D, Glypican-4, Insulin, Type 2 diabetes

## Abstract

**Background:**

Glycosylphosphatidylinositol-specific phospholipase D (GPLD1) is responsible for cleaving membrane-associated glycosylphosphatidylinositol (GPI) molecules, which is affected by diabetes. We aimed to examine the effect of 14 weeks treadmill running on serum GPLD1 levels and its association with glycemic indexes and serum glypican-4 (GPC-4), a novel GPI-anchored adipokine, in streptozotocin-nicotinamide-induced diabetic rats.

**Methods:**

Thirty-six male Wister rats were randomly divided into three groups of twelve animals each, involving sedentary control (SC), sedentary diabetic (SD), and trained diabetic (TD) groups. The diabetes was induced through intraperitoneal injection of 120 mg/kg nicotinamide 15 min prior to intraperitoneal injection of 65 mg/kg streptozotocin in SD and TD groups. The TD group was exercised on a treadmill for 60 min/days, 5 days/wk at 26 m/min, and zero grade for 14 weeks. Following the experiment period, blood samples were taken from all animals and analyzed for experimental indexes via sandwich ELISA.

**Results:**

Exercise training caused a significant decrease in the elevated blood glucose levels and a significant increase in the lowered blood insulin levels in TD rats (both p < 0.001). Glucose tolerance of TD rats significantly improved following experimental protocol, as indicated by OGTT (p < 0.001). Experimental diabetes significantly increased serum GPLD1 levels (p < 0.001), while exercise training significantly decreased its levels (p < 0.001). Serum GPLD1 levels correlated directly with glycemic indexes involving FBS, 2hOGTT, and AUC of glucose (r = 0.80, r = 0.79, r = 0.79, respectively, all p < 0.001) and inversely with serum insulin levels (r = − 0.83, p < 0.001). There were no significant differences in serum GPC-4 levels among groups, and no association with GPLD1 alteration.

**Conclusions:**

Sedentary diabetic rats have higher circulating GPLD1 compared to controls, which can be reversed by exercise training and is associated with modifying in glycemic and insulin profile.

## Background

Glycosylphosphatidylinositol-specific phospholipase D (GPLD1, also called GPI-PLD) is a 110- to 120-kDa *N*-glycosylated amphiphilic protein abundant in mammalian serum, where it associates with high-density lipoproteins (HDL) [1]. It is an 815-amino acid enzyme expressed in numerous tissues and cells [2]. Liver and pancreatic islets are two likely sources of GPLD1; however, studies have demonstrated that brain, kidney, muscle, immunocytes, and inflammatory cells may also be sources [3]. Most glycosylphosphatidylinositol (GPI)-anchored proteins have been shown to be cleaved by GPI phospholipases. Recently, GPLD1 has been shown to be a strictly specific enzyme for GPI anchors [4]. This enzyme can play a role in glucose metabolism in skeletal muscle, as a GPLD1 has been implicated in translocation of the glucose transporter 4 transporter in a cell-free rat skeletal muscle system [5]. In addition, it is possible that by hydrolyzing the GPI anchors of some inflammatory membrane proteins and upregulating macrophage cytokine expression, GPLD1 may play an important role in inflammation and in the pathogenesis of diabetes [3]. Moreover, its expression by insulinoma cell lines and pancreatic islet preparations has lead to the speculation that it may be involved in diabetes or is affected by different diabetic states [6]. Interestingly, accumulating evidence has been indicated that GPLD1 levels increased in circulating compartment following the onset of diabetes induction and insulin resistance both in rats and humans, respectively, suggesting its notable association with diabetes-induced impairments [7, 8].

GPI-anchored proteins, such as heparan sulfate proteoglycans including glypican family (GPC-1 to GPC-6), are tethered to the surface of mammalian cells via the lipid GPI and have been implicated in many important cellular functions including cell adhesion, cell signaling, and immune regulation [9]. It has been suggested that GPLD1 has a role in cell signaling, whereby GPLD1 cleaves GPIs on the external membrane generating inositolphosphoglycan moiety which appears to act as second messengers in a variety of hormone and cytokine-mediated events [7]. Notably, the cell surface glypican-4 (GPC-4) was shown to be released primarily by adipocytes, following proteolytic cleavage of the GPI anchor by lipases, such as the insulin-regulated GPLD1 [10]. GPC-4, as a potential ligand and positive modulator of insulin receptor (IR), enhances insulin signaling by binding to IR α subunits and facilitating the insulin-induced formational change of IR [11]. Recently, it has been focused on circulating levels of GPC-4 in different glucose metabolism status due to its notable alterations in subjects from normal glucose tolerance to patients with diabetes [12].

Exercise training has long been recognized as a nonpharmacological method in the treatment regimen of diabetes, which results in a variety of physiological and metabolic adaptations [13]. It has been shown to stimulate glucose uptake, improve glycemic control and insulin action, and decrease hyperglycemia in insulin-resistant states, including type 2 diabetes, via various signaling processes and molecular mechanisms [14]. In particular, aerobic exercise over the long term is a well-established training type to modify glycemic indexes by amending of proteins involved in the pathogenesis of diabetes [15]. As seen above, GPLD1 has a notable role in glucose metabolism and pathogenesis of diabetes. It has been reported that insulin activates GPLD1 and induces GPI hydrolysis by this enzyme [16]. It is considerable that endurance exercise with moderate intensity has been suggested to induce beneficial effects, at least in part, by the improvement in insulin profile in diabetic rats [17]. Although accumulating studies have been shown a variety of therapeutic mechanisms of exercise training in diabetes, a paucity of knowledge exists regarding the exercise-induced regulation and function of GPLD1 in serum. Hence, to test the hypothesis that anti-diabetic effects of exercise training may alter GPLD1, and it may be involved in exercise-induced adaptations in diabetes, we examined 14 weeks treadmill running on serum GPLD1 levels and its association with insulin and glycemic status in streptozotocin-nicotinamide-induced diabetic rats. A second aim was to determine whether any changes in serum GPLD1 are accompanied with a significant change in serum GPC-4 following the study approach.

## Materials and methods

### Animal care and feeding

The trial procedures were done on thirty-six male Wister albino rats aged 3 months and weighing 210 to 230 g, which conducted in exact under institutional guidelines and confirmed by the ethical committee of laboratory animals care at Bu-Ali Sina University (BASU), Hamedan, Iran. The rats were maintained in the Animal House Center, Department of Sport Science of Bu-Ali Sina University and housed 6 per cage (46 L in volume, 24.5 × 15 × 8 in) with a 12:12 h light–dark cycle. During all of the procedures, temperature and humidity were adjusted to 22 °C ± 2 °C and 55% ± 5%, respectively. The rats were given rodent chow pellets and water ad libitum.

### Diabetogenicity protocol

All rats were familiarized with the animal exercise physiology laboratory conditions for 1 week [18]. Following this phase, the rats were weight-matched and randomly assigned into 1 of 3 experiment-equal groups (n = 12): the sedentary control (SC), sedentary diabetic (SD), and trained diabetic (TD) groups. The diabetogenicity in SD and TD groups were done according to streptozotocin–nicotinamide (STZ–NA) protocol. Hence, the rats were received 120 mg/kg NA (Sigma-Aldrich, St. Louis, Missouri, United States) dissolved in normal saline buffer, via the intraperitoneal injection. Following 15 min, 65 mg/kg STZ (Sigma-Aldrich, St. Louis, Missouri, United States) dissolved in 0.1 M citrate buffer, pH 4.5, was injected intraperitoneally. The rats were fasted overnight before the induction of diabetes. Following 72 h, blood samples was obtained from a small nick in the tail of rats and fasting blood glucose (FBS) concentration measured using a glucometer (Acuu-Chek Active, Roche, Germany). The induction of diabetes was confirmed if the FBS was beyond of 126 mg/dL [19]. The control groups remained sedentary, whereas the training group performed a 14-week period of regular moderate exercise training consist of running on a motor-driven treadmill.

### Exercise training protocol

Animals of the training group underwent running on the motor-driven treadmill (5 days/week, for 14 weeks). The inclination of the treadmill was set at zero, and the target intensity of exercise training was determined as moderate through the speed of the treadmill with 26 m/min for 60 min/day [20]. Initially, in the first step, treadmill training began by familiarizing the rats with the apparatus, which the speed of running and time course was regarded 8 m/min and 15 min a day, respectively. Then, in the second step, the load of exercise training gradually intensified every week by adding 2 m/min and 5 min to the speed of running and time course, respectively. The second step elongated from week 2 to week 10. Finally, in the third step, the speed of running and time course reached to 26 m/min and 60 min a day, respectively. The third step continued for the last 4 weeks, and its load steadily stayed constant. Based on the triplet periods, each training session began with a 5 to 10 min warm-up during which the speed was progressively increased from zero to the target training speed. At the end of each training session, a reverse process performed for cool-down. Running animals were closely monitored to ensure their safety and training compliance.

### Oral glucose tolerance test (OGTT)

Following the completion of trial procedures and about 24 h subsequent to the last exercise training session, the oral glucose tolerance test (OGTT) was performed on overnight-fasted rats. All groups of rats were orally received 2 g/kg of a 100 mg/ml glucose solution in distilled water by gavage. Bit blood volumes were obtained through a needle prick to the tip of the tail at 0 (prior to treatment), 30, 60 and 120 min following glucose loading. The blood glucose concentrations were calculated by applying a glucometer (Acuu-Chek Active, Roche, Germany). Then, the trapezoidal method was applied for the calculation of the area under the curves (AUC) of glucose [21].

### Serum analysis

Following the completion of trial procedures and about 48 h subsequent to the last exercise training session (14th week), all groups of rats were anesthetized by means of ether. Prior to blood sampling, the rats were fasted overnight. Following perfect anesthesia and splitting of rats’ abdomen, the blood samples were collected from the inferior vena cava and inserted into the serum gel tubes. Then, the centrifugations of samples were performed for 10 min at 3000 rpm. The collected serum was set into the micro-tubes and kept at − 36 °C until the analyses of circulating parameters.

The technique of sandwich enzyme-linked immunosorbent assay (ELISA) was applied for quantification of endogenous serum Glycosylphosphatidylinositol-specific phospholipase D (GPLD1) levels via a rat Glycosylphosphatidylinositol-specific phospholipase D ELISA kit (Rat Phosphatidylinositol-Glycan-Specific Phospholipase D (GPLD1) ELISA Kit, ZellBio GmbH, Ulm, Germany), based on the manufacturers’ protocol. The coefficient of variation of this kit was 10%. In a similar method, the technique of sandwich ELISA was applied for quantification of endogenous serum glypican-4 (GPC-4) levels via a rat Glypican-4 ELISA kit (Rat Glypican-4 (GPC-4) ELISA Kit, ZellBio GmbH, Ulm, Germany), based on the manufacturers’ protocol. The coefficient of variation of this kit was 10%. The levels of insulin in serum were quantified using a rat insulin ELISA kit (Mercodia Rat Insulin ELISA Kit, Uppsala, Sweden), based on the manufacturers’ protocol. The coefficient of variation of this kit was 5.1%. The levels of glucose in serum were quantified using an enzymatic, colorimetric method (glucose oxidase amino antipyrine; Pars Azmoun, Tehran, Iran) based on the manufacturers’ protocol. The coefficient of variation of the method was 1.3%. All samples were analyzed in duplicate. The following formula was used for the measurement of body mass index (BMI) [22]: BMI = body weight (g)/the squares of the length (cm).

### Statistical analysis

The software of SPSS 19.0 (SPSS, IBM, Chicago, Illinois, United States) was applied for calculation of all statis-tical analysis. The normal distribution of data was tested using the Kolmogorov–Smirnov (K–S) test. Based on the K–S test, the distribution of body weight, length, body mass index, and GPC-4 were normal, and the other indexes were abnormal. Results were expressed as the mean ± standard deviation (SD). Comparisons between groups were analyzed either by one-way analysis of variance (ANOVA) with post hoc analysis with Tukey test or Kruskal–Wallis test for normally and abnormally distributed data, respectively. Pearson’s or Spearman’s correlations were used to examine the relationship between variables with normally and abnormally distributed data, respectively. The statistical significance level was determined at p < 0.05.

To assessment of effect sizes, Partial eta-squared statistic was calculated for ANOVA tests. The small, medium, and large effects were considered in values of $\eta_{p}^{2}$ equal to 0.01- < 0.06, 0.06 - < 0.14, and ≥ 0.14, respectively. In addition, Eta squared based on the H-statistic ($\eta^{ 2}_{H}$)was calculated for Kruskal–Wallis tests. The small, medium, and large effects were considered in values of $\eta^{ 2}_{H}$ equal to 0.01 to  < 0.06, 0.06 to  < 0.14, and ≥ 0.14, respectively [23].

## Results

### Physical measurments

The physical characteristics of the rats are listed in Table 1. At the end of the experiment, following 14 weeks, the results revealed that body weights and body mass index (BMI) were not significantly different among groups (*p* = 0.141, *p* = 0.732, respectively) (Table 1). The effect size of differences in body weights and body mass index (BMI) was medium ($\eta_{p}^{2}$ = 0.11, $\eta_{p}^{2}$ = 0.1, respectively) (Table 2).

**Table 1:** The morphometric, metabolic, hormonal and biochemical measurements of the rats following 14 weeks in the groups

Variables	SC	SD	TD
Body weight (g)	231 ± 17	235 ± 24	211 ± 44
Length (cm)	22 ± 0.74	22 ± 0.85	21 ± 1.5
BMI	0.477 ± 0.02	0.489 ± 0.07	0.474 ± 0.03
FBS (mmol/L)	6.14 ± 0.58	21.72 ± 1.24^a^	16.92 ± 3.35^a, b^
2hOGTT (mmol/L)	6.72 ± 0.64	44.21 ± 2.61^a^	30.61 ± 2.21^a, b^
AUC glucose (mmol/L × 120 min)	873 ± 78	4937 ± 264^a^	3703 ± 115^a, b^
FIns (μU/mL)	31.56 ± 3.4	8.73 ± 3.42^a^	14.7 ± 3.97^a, b^
GPLD1 (ng/mL)	33.41 ± 3.65	48.78 ± 2.78^a^	36.37 ± 4.06^b^
GPC-4 (ng/mL)	2.13 ± 0.19	2.3 ± 0.33	2.07 ± 0.37

Values are expressed as mean ± SD, and n = 12 for all groups

*SC* sedentary control group, *SD* sedentary diabetic group, *TD* trained diabetic group, *BMI* body mass index, *FBS* fasting blood sugar, *FIns* fasting serum insulin, *2hOGTT* 2 h post-glucose load blood glucose, *AUC* glucose, area under the curve of glucose, *GPLD1* glycosylphosphatidylinositol-specific phospholipase D, *GPC-4* glypican-4.

^a^*p *< 0.001 vs. SC; ^b^*p *< 0.001 vs. SD

**Table 2:** The effect size of research assessments

Indexes of ES	Variables	Values of ES	Magnitude of ES
Partial eta-squared statistic $(\eta_{p}^{2})$	Body weight	0.11	Medium
	BMI	0.1	Medium
	GPC-4	0.12	Medium
Eta squared based on the H-statistic ($\eta^{ 2}_{H}$)	FBS	0.87	Large
	2hOGTT	0.88	Large
	AUC glucose	0.88	Large
	FIns	0.83	Large
	GPLD1	0.68	Large

*BMI* body mass index, *ES* effect size, *FBS* fasting blood sugar, *FIns* fasting serum insulin, *2hOGTT* 2 h post-glucose load blood glucose, *AUC* glucose, area under the curve of glucose, *GPLD1* glycosylphosphatidylinositol-specific phospholipase D, *GPC-4* glypican-4

### Glycemic measurments

Serum glucose, 2hOGTT, and AUC of glucose of the rats are listed in Table 1. As expected, serum glucose levels were observed to be significantly high in the SD group (p < 0.001) as compared to the SC group. Conversely, the low levels of serum glucose were found in the TD group (p < 0.001) as compared to the SD group, although the serum glucose in the TD group did not return to the normal levels in the SC group (Table 1). The effect size of differences in serum glucose was large ($\eta_{H}^{2}$ = 0.87) (Table 2). There were significant differences in 2hOGTT and AUC of glucose among groups (Table 1). Consistent with the data of serum glucose levels, 2hOGTT and AUC of glucose were significantly higher in the SD group (both *p *< 0.001) as compared to the SC group. The lower levels of 2hOGTT and AUC of glucose were found in the TD group as compared to the SD group (both *p *< 0.001). The higher levels of 2hOGTT and AUC of glucose were found in the TD group as compared to the SC group (both *p *< 0.001). The effect size of differences in 2hOGTT and AUC of glucose was large (both $\eta^{ 2}_{H}$  = 0.88) (Table 2). The results of OGTT at different time intervals are listed in Table 3. The serum glucose levels at 0, 30, 60, and 120 min in the SD group were significantly higher than those in the SC group (all *p *< 0.001). However, the serum glucose levels at 0, 30, 60, and 120 min in TD group were significantly lower than the SD group (all *p *< 0.001) but higher than the SC group (all *p *< 0.001) (Table 3). The effect size of differences in OGTT at 0, 30, and 60 min was large ($\eta^{ 2}_{H}$ = 0.81, $\eta^{ 2}_{H}$ = 0.88, $\eta^{ 2}_{H}$ = 0.88, respectively).

**Table 3:** The results of oral glucose tolerance test (OGTT) following 14 weeks in the groups

Groups	Blood glucose levels (mmol/L)			
	0 min	30 min	60 min	120 min
SC	5.96 ± 0.41	8.33 ± 0.81	7.37 ± 0.72	6.72 ± 0.64
SD	22.32 ± 1.68^a^	34.63 ± 1.95^a^	49.70 ± 2.92^a^	44.21 ± 2.61^a^
TD	19.39 ± 1.13^a, b^	26.43 ± 1.16^a, b^	37.81 ± 1.68^a, b^	30.61 ± 2.21^a, b^

Values are expressed as mean ± SD, and n = 12 for all groups

*SC* sedentary control group, *SD* sedentary diabetic group, *TD* trained diabetic group

^a^*p *< 0.001 vs. SC; ^b^*p *< 0.001 vs. SD

### Hormonal and biochemical measurments

**Fig. 1:**
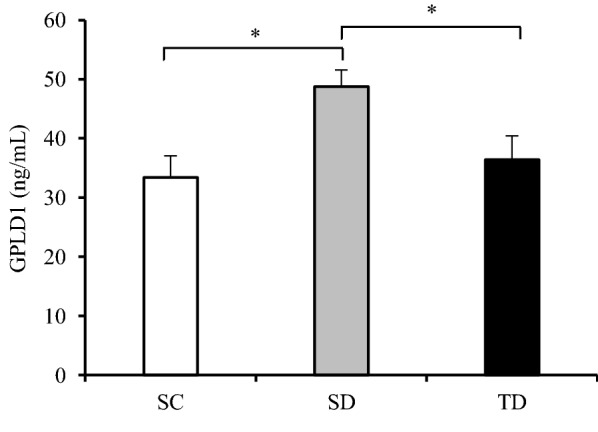
Serum GPLD1 following 14 weeks in the groups. SC, sedentary control group; SD, sedentary diabetic group; TD, trained diabetic group; GPLD1, glycosylphosphatidylinositol-specific phospholipase D. Data are expressed as mean ± SD (n = 12). * *p *< 0.001 vs. SD group

Serum insulin, GPLD1, and GPC-4 of the rats are listed in Table 1. Serum insulin levels were significantly different among groups (Table 1). Serum insulin levels were significantly low in the SD group (*p *< 0.001) as compared to the SC group. On the contrary, serum insulin levels were significantly higher in the TD group (*p *< 0.001) as compared to the SD group, although the serum insulin levels in the TD group did not reach the normal levels in the SC group (Table 1). The effect size of differences in serum insulin was large ($\eta^{ 2}_{H}$ = 0.83) (Table 2). Noteworthy, there were significant differences in serum GPLD1 levels among groups (Table 1). Serum GPLD1 levels were significantly high in the SD group (p < 0.001) as compared to the SC group. However, following 14 weeks of exercise training, the serum GPLD1 levels of the TD group were negatively affected by exercise training and were significantly lower (p < 0.001) as compared with the SD group. The effect size of differences in serum GPLD1 was large ($\eta^{ 2}_{H}$ = 0.68) (Table 2). Interestingly, there was no significant difference in serum GPLD1 levels between the TD and SC groups (Fig. 1). Then, no significant differences in serum GPC-4 levels were observed among groups (p = 0.11), although the mean serum GPC-4 levels of TD group decreased compared to the SC and SD groups, it was not statistically significant (Table 1). The effect size of differences in serum GPC-4 was medium ($\eta_{p}^{2}$ = 0.12) (Table 2).

**Fig. 2:**
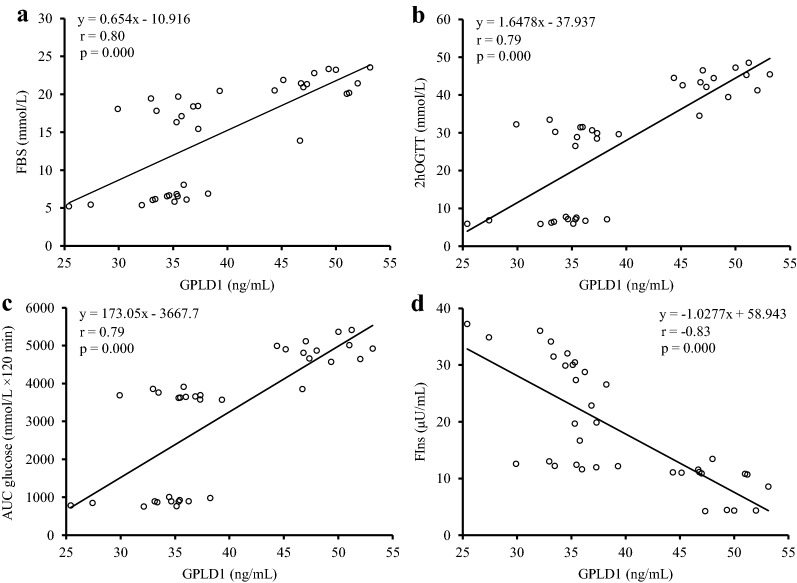
Spearman’s rank correlation coefficient between serum GPLD1 with FBS **a**, 2hOGTT **b**, AUC glucose **c**, and FIns **d** following 14 weeks in the groups. FBS, fast blood sugar; FIns, fasting serum insulin; 2hOGTT, 2 h post-glucose load blood glucose; AUC glucose, area under the curve of glucose; GPLD1, glycosylphosphatidylinositol-specific phospholipase D

We further analyzed the association between serum GPLD1 levels with glycemic parameters and circulating insulin levels because GPLD1 is well-known to be associated with diabetes. Fig. 2 shows the correlation between serum GPLD1 levels with serum glucose levels, 2hOGTT, AUC of glucose, and serum insulin levels. Notably, serum GPLD1 levels were positively correlated with serum glucose levels (Fig. 2a), 2hOGTT (Fig. 2b) and AUC of glucose (Fig. 2c) (r = 0.80, r = 0.79 and r = 0.79, respectively, all *p *< 0.001). Besides, serum GPLD1 levels were negatively correlated with serum insulin levels (Fig. 2d) (r = − 0.83, *p *< 0.001). However, there was no significant association between serum GPLD1 levels and serum GPC-4 levels (r = 0.31, *p* = 0.068). Similarly, no significant association was found between serum GPC-4 levels and above mentioned glycemic parameters and circulating insulin.

## Discussion

Diabetes mellitus is a growing public health problem worldwide, which could be improved through behavioral modification such as physical activity [24]. Exercise training orchestrates numerous metabolic adaptations in different tissues and can improve whole-body metabolism and glucose homeostasis. It is widely accepted that exercise training induced-adaptations are associated with alteration in protein content and enzyme activities [25]. The elucidating of these molecular mechanisms provides a more comprehensive conceptual basis for the understanding of the metabolic homeostasis, which how exercise training could exert its beneficial effects. GPLD1, the focus attention of our study, is an enzyme abundant in serum, which has been associated with different glucose metabolism status and diabetes.

In the present study, sedentary diabetic rats showed a significant increase in serum GPLD1 levels as compared with corresponding rats in the sedentary control group. In line with our finding, several studies have reported that circulating GPLD1 levels increased in patients with type 1 diabetes, STZ-induced diabetic rats [7], nonobese diabetic mice, STZ-induced diabetic CD-1 mice [1] and high-fructose diet-induced diabetic mice [26] as compared to the controls. Further studies have demonstrated that GPLD1 expression increased in the liver of diabetic mice [1], skeletal muscle of ob/ob mice [27], and isolated islet β cells of ob/ob mice [28]. However, the main finding of the present study was a lower serum GPLD1 levels in the trained diabetic group (Fig. 1), indicating that serum GPLD1 respond to 14 weeks exercise training in diabetic rats. It is also worth noting that serum GPLD1 levels in trained diabetic rats statistically restored to the normal levels in the sedentary control group. Hence, circulating GPLD1 levels showed a bell-shaped profile from normal to a sedentary diabetic, and to a training diabetic state.

The mechanism(s) by which exercise training affects serum GPLD1 levels has not been investigated at this time. However, it has been suggested that serum levels of GPLD1 may be regulated by a number of different hormones or metabolites involving circulating insulin levels, hyperglycemia, oxidative stress, and inflammation [1, 29]. In the present study, decreased GPLD1 was accompanied by decreased blood glucose concentration and decreased glucose tolerance as indicated by the OGTT. Consistent with this association, Deeg et al. [1] found that with the onset of hyperglycemia, two to fivefold increase over nondiabetic levels, GPLD1 serum activity and liver mRNA increased in both nonobese diabetic mice and STZ-induced diabetic CD-1 mice. In addition, Bowen et al. [28] showed that incubation of high glucose increased cellular GPLD1 activity and mRNA levels in isolated rat islets and βTC3, a murine β-cell line. These findings suggest that glucose and especially hyperglycemia play an important role both in the synthesis and release of GPLD1. Considering the strong association of decreased serum GPLD1 with FBS, 2hOGTT, and AUC of glucose in OGTT (Fig. 2a–c), it is likely that exercise-induced improvement in the glycemic profile of trained diabetic rats is a mechanism, which caused a decline in circulating GPLD1 levels.

Our observation that decreased serum GPLD1 levels are associated with increased serum insulin levels (Fig. 2d) raises the possibility of a link between insulin and the regulation of this enzyme. In support of this possibility, it has been reported that the treatment of diabetic mice with insulin corrected the hyperglycemia along with a rapid and complete return of serum GPLD1 activity and liver mRNA [1]. Similarly, Schofield et al. [7] showed that insulin decreases circulating GPLD1 levels in rats and humans with diabetes by inhibiting GPLD1 synthesis in the liver. Besides, it has been suggested that increased GPLD1 may be part of the compensatory response to increased insulin demand [30]. Hence, increased serum insulin levels in trained diabetic rats may partly decrease the requirement of this compensatory response via GPLD1. From the other point of view, O’Brien et al. [29] indicated that in vitro upregulation of macrophage GPLD1 expression by exposure to H_2_O_2_ suggests that oxidants may be important regulators of GPLD1 expression in inflammation. It has been well documented that exercise training improves antioxidant systems [17] and anti-inflammatory responses [31] both in the normal and diabetic state. However, further experiments are required to examine the potential antioxidant and anti-inflammatory roles of exercise training in the regulation of GPLD1 in models of diabetes.

GPC-4, a novel adipokine, is ordinarily a membrane-bound hormone due to a GPI anchor, and GPLD1 has been suggested to cleave its anchor, releasing it into the circulation [10]. Indeed, in proteoglycans, GPC-4 is the first-identified adipokine that associates with diabetes via increasing insulin sensitivity [32]. It has been shown that activation of peroxisome proliferators-activated receptor γ (PPARγ), a ligand-activated transcription factor belonging to nuclear hormone receptors of steroid receptor superfamily [33], increases the GPC-4 expression in diabetic mice [10, 34]. In addition, endurance exercise training with low to moderate intensity has been reported to induce PPARγ activation in both mice and human [35, 36]. Hence, the protocol of exercise training with the progressive process from low to moderate intensity, as target intensity, was considered for the present study. Based on the findings of the present study, no significant change was observed in serum GPC-4 levels among groups. Although a slight decline in serum GPC-4 levels of trained diabetic rats was accompanied by change style in serum GPLD1 levels, their association was not statistically significant. The effect of exercise training on GPC-4 is poorly investigated as well as comparable studies are lacking. Consistent with the finding of the present study, Yoo et al. [37] reported that no significant change in circulating GPC-4 levels observed in nondiabetic obese women following 3-month combined aerobic and resistance exercise training. It has been suggested that circulating GPC-4 levels were positively associated with BMI in subjects with different glucose metabolism status [12, 38]. Hence, serum GPC-4 levels are closely associated with obesity-related parameters, implying the relatively greater importance of body fat distribution on circulating GPC-4 levels [12]. However, in this study nonobese rats were used, and there was no difference in BMI among groups following the experimental period. The absence of serum GPC-4 alteration in the present study may associate with the absence of BMI difference in the groups. Taken together, it seems that serum GPC-4 does not play a significant role in long-term exercise adaptation. However, we cannot refuse the acute effects of exercise training on serum GPC-4. Hence, further investigation of the acute effects of exercise training on the turnover of GPC-4 is recommended in different glucose metabolism states and diabetes.

The main limitation of the present study is that no trained control group was included. This research design is a three-group design with a post-test measurement, not a pretest–posttest design. In the present study design, the sedentary control (SC) group has been considered as the baseline level for the research factors. Regarding its limitations, this experiment was purposed as a primary study of response to the exercise training, and its findings cleared the alteration of serum GPLD1 levels in trained and sedentary physical conditions. However, the hopeful findings will deserve a larger study in larger sample sizes and more measurements.

## Conclusions

The present study revealed that experimental diabetes increased circulating GPLD1 levels in rats. Simultaneously, we studied the endurance exercise training as an approach to investigate the alteration and regulation of circulating GPLD1. The findings showed a difference in circulating GPLD1 levels in normal, sedentary diabetic, and trained diabetic rats as well as a significant association between circulating GPLD1 levels with glycemic parameters and circulating insulin levels. Our results demonstrated for the first time that endurance exercise training restored circulating GPLD1 concentrations in diabetic rats to normal levels. The findings also indicated that alterations in circulating GPLD1 concentrations did not accompany by circulating GPC-4 concentrations. Further experiments are required to determine the effects of exercise training on the regulation and function of GPLD1 in diabetes.

## Data Availability

The datasets used in the present study are available from the corresponding author on reasonable request.

## Abbreviations

2hOGTT: 2 h post-glucose load blood glucose
AUC glucose: Area under the curve of glucose
BMI: Body mass index
FBS: Fasting blood sugar
FIns: Fasting serum insulin
GPI: Glycosylphosphatidylinositol (anchor)
GPLD1: Glycosylphosphatidylinositol-specific phospholipase D
GPC-4: Glypican-4
NA: Nicotinamide
OGTT: Oral glucose tolerance test
STZ: Streptozotocin
